# Procedural and quality assessment data on catheter ablation for fascicular ventricular tachycardia

**DOI:** 10.1016/j.dib.2018.11.027

**Published:** 2018-11-13

**Authors:** Antonio Creta, Anthony W. Chow, Simon Sporton, Malcolm Finlay, Nikolaos Papageorgiou, Shohreh Honarbakhsh, Gurpreet Dhillon, Adam Graham, Kiran HK Patel, Mehul Dhinoja, Mark J. Earley, Ross J. Hunter, Martin Lowe, Edward Rowland, Oliver R. Segal, Vito Calabrese, Danilo Ricciardi, Pier D. Lambiase, Richard J. Schilling, Rui Providência

**Affiliations:** a Barts Heart Centre, St. Bartholomew׳s Hospital, London, United Kingdom; b Campus Bio-Medico University of Rome, Rome, Italy; c Northwick Park Hospital, London, United Kingdom

## Abstract

Data presented in this article are supplementary materials to our article entitled “Catheter Ablation for Fascicular Ventricular Tachycardia: A Systematic review” (Creta et al., 2018). The current article provides additional procedural data regarding the catheter ablation for fascicular ventricular tachycardia (FVT) performed in the patients enrolled in our analysis. Furthermore, we provide data regarding the quality assessment of the studies included in our systematic review.

**Specifications table**

**Table:** 

Subject area	*Medicine*
More specific subject area	*Cardiology- catheter ablation*
Type of data	*Tables*
How data were acquired	*Review and quality assessment of available literature*
Data format	*Analyzed and raw materials*
Experimental factors	*Statistical analyses with Comprehensive Meta-Analysis software (Version 2)*
Experimental features	*Systematic review*
Data source location	*PubMed, EMBASE and Cochrane database*
Data accessibility	*Data are with this article*
Related research article	Creta A, Chow A, Sporton S, Finlay M, Papageorgiou N, Honarbakhsh S, Dhillon G, Graham A, Patel K, Dhinoja M, Earley MJE, Hunter RJ, Lowe M, Rowland E, Segal OR, Calabrese V, Ricciardi D, Lambiase PD, Schilling RJ, Providência R. Catheter ablation of fascicular ventricular tachycardia: a systematic review. [1]

**Value of the data**

• These data can be used for defining the scientific quality of the most relevant published series of patients undergoing catheter ablation for FVT.
• These data also provide useful information for guiding the ablation strategy for FVT, by defining the timing of the pre-Purkinje potentials at the successful ablation site and the incidence of fascicular block after ablation.

## 1 Data

The data presented in this article are supplementary material to our systematic review on catheter ablation for FVT [1].

Table S1 shows the results of the quality assessment for case series studies using the National Heart, Lung, and Blood Institute scale [2].

Table S2 shows supplementary procedural data on catheter ablation for FVT. We report the earliest interval between pre-Purkinje potentials and QRS during tachycardia at the ablation site. Furthermore, we provide data regarding incidence of fascicular block after catheter ablation.

## 2 Experimental design, materials and methods

We performed a systematic electronic search of the published studies including patients who underwent catheter ablation for FVT [1]. Thirty-eight studies were selected. Quality assessment was performed for each study by two independent reviewers (AC and RP) using the National Heart, Lung, and Blood Institute Quality Assessment Tool for Case Series Studies [2]. The third author (AWC) intervened to resolve disputes in case of disagreement of the two principal reviewers regarding the classification of a study. The procedural data from each study were also analysed, with a focus on the interval between pre-Purkinje potentials and QRS complex during tachycardia at the ablation site and the incidence of fascicular block after catheter ablation.
