# Supplementary material for: Procedural and quality assessment data on catheter ablation for fascicular ventricular tachycardia
Source: Data Brief. 2018 Nov 13;21:2376–8. doi: 10.1016/j.dib.2018.11.027 (PMC6280585; doi:10.1016/j.dib.2018.11.027)
Supplement: Supplementary file 1 — Supplementary material [file mmc1.docx]

DECLARATION OF INTEREST

RJS has had research agreements and speaker fees from Abbott, Medtronic, Boston Scientific and Biosense Webster. PDL has received educational grants from Medtronic and Boston Scientific. MF has received speaker fees from Biotronik and Medtronic and owns stocks of Epicardio ltd. All other authors have reported that they have no relationships relevant to the contents of this paper to disclose.
