# Supplementary material for: Procedural and quality assessment data on catheter ablation for fascicular ventricular tachycardia
Source: Data Brief. 2018 Nov 13;21:2376–8. doi: 10.1016/j.dib.2018.11.027 (PMC6280585; doi:10.1016/j.dib.2018.11.027)
Supplement: Supplementary file 2 — Supplementary material [file mmc2.docx]

**S-Table 1.** Quality Assessment Scale for Case Series Studies – National Heart, Lung, and Blood Institute [2]

| **Criteria** | **Studies** | | | | | | | | |
| --- | --- | --- | --- | --- | --- | --- | --- | --- | --- |
|  | Ma 2017  [38] | Luo 2017 [24] | Guo 2017 [13] | Zhan 2016  [25] | Liu Q 2016 [8] | Chen 2016 [19] | Liu Y 2015 [7] | Letsas 2015 [39] | Gopi 2015 [14] |
| 1. Was the study question or objective clearly stated? | Yes | Yes | Yes | Yes | Yes | Yes | Yes | Yes | Yes |
| 2. Was the study population clearly and fully described, including a case definition? | Yes | Yes | Yes | Yes | Yes | Yes | Yes | Yes | Yes |
| 3. Were the cases consecutive? | NR | Yes | Yes | Yes | Yes | Yes | Yes | Yes | NR |
| 4. Were the subjects comparable? | No | No | No | No | No | No | No | No | No |
| 5. Was the intervention clearly described? | Yes | Yes | Yes | Yes | Yes | Yes | Yes | Yes | Yes |
| 6. Were the outcome measures clearly defined, valid, reliable, and implemented consistently across all study participants? | Yes | Yes | Yes | Yes | Yes | Yes | Yes | Yes | Yes |
| 7. Was the length of follow-up adequate? | Yes | Yes | Yes | Yes | Yes | Yes | Yes | Yes | Yes |
| 8. Were the statistical methods well-described? | Yes | Yes | Yes | Yes | Yes | Yes | Yes | No | No |
| 9. Were the results well-described? | Yes | Yes | Yes | Yes | Yes | Yes | Yes | Yes | Yes |

| **Criteria** | **Studies** | | | | | | | | |
| --- | --- | --- | --- | --- | --- | --- | --- | --- | --- |
|  | Fishberger 2015 [22] | Talib 2015  [17] | Liu XY 2014 [26] | Suzuki 2014 [48] | Collins  2013 [15] | Kataria 2013 [20] | Park 2012  [40] | Wissner  2012 [41] | Chu 2012 [27] |
| 1. Was the study question or objective clearly stated? | Yes | Yes | Yes | Yes | Yes | Yes | Yes | Yes | Yes |
| 2. Was the study population clearly and fully described, including a case definition? | Yes | Yes | Yes | Yes | Yes | Yes | Yes | Yes | Yes |
| 3. Were the cases consecutive? | NR | Yes | Yes | NR | NR | Yes | No | Yes | Yes |
| 4. Were the subjects comparable? | No | No | No | No | No | No | No | No | No |
| 5. Was the intervention clearly described? | Yes | Yes | Yes | Yes | Yes | Yes | Yes | Yes | Yes |
| 6. Were the outcome measures clearly defined, valid, reliable, and implemented consistently across all study participants? | Yes | Yes | Yes | Yes | Yes | Yes | Yes | Yes | Yes |
| 7. Was the length of follow-up adequate? | Yes | Yes | Yes | Yes | Yes | Yes | Yes | Yes | Yes |
| 8. Were the statistical methods well-described? | No | Yes | Yes | No | Yes | Yes | Yes | No | Yes |
| 9. Were the results well-described? | Yes | Yes | Yes | Yes | Yes | Yes | Yes | Yes | Yes |

| **Criteria** | **Studies** | | | | | | | | |
| --- | --- | --- | --- | --- | --- | --- | --- | --- | --- |
|  | He 2011  [28] | Shin 2010  [21] | Ma  2006 [42] | Magalhaes 2006 [16] | Lin 2005 [43] | Arya 2004  [29] | Topilski 2004 [18] | Gupta 2002 [44] | Ouyang  2002 [30] |
| 1. Was the study question or objective clearly stated? | Yes | Yes | Yes | Yes | Yes | Yes | Yes | Yes | Yes |
| 2. Was the study population clearly and fully described, including a case definition? | Yes | Yes | Yes | Yes | Yes | Yes | Yes | Yes | Yes |
| 3. Were the cases consecutive? | Yes | No | Yes | NR | Yes | Yes | Yes | NR | Yes |
| 4. Were the subjects comparable? | No | No | No | No | No | No | No | No | No |
| 5. Was the intervention clearly described? | Yes | Yes | Yes | Yes | Yes | Yes | Yes | Yes | Yes |
| 6. Were the outcome measures clearly defined, valid, reliable, and implemented consistently across all study participants? | Yes | Yes | Yes | Yes | Yes | Yes | Yes | Yes | Yes |
| 7. Was the length of follow-up adequate? | Yes | Yes | Yes | Yes | Yes | Yes | Yes | Yes | Yes |
| 8. Were the statistical methods well-described? | Yes | Yes | No | No | No | Yes | Yes | Yes | Yes |
| 9. Were the results well-described? | Yes | Yes | Yes | Yes | Yes | Yes | Yes | Yes | Yes |

| **Criteria** | **Studies** | | | | | | | | |
| --- | --- | --- | --- | --- | --- | --- | --- | --- | --- |
|  | Aiba 2001  [31] | Miyauchi 2000 [32] | Nogami 2000 [33] | Tsuchiya 1999 [34] | Nogami 1998 [35] | Bennett 1997 [47] | Wen 1997  [45] | Katristis 1996 [23] | Zardini 1995 [36] |
| 1. Was the study question or objective clearly stated? | Yes | Yes | Yes | Yes | Yes | Yes | Yes | Yes | Yes |
| 2. Was the study population clearly and fully described, including a case definition? | Yes | Yes | Yes | Yes | Yes | Yes | Yes | Yes | Yes |
| 3. Were the cases consecutive? | Yes | Yes | Yes | Yes | Yes | Yes | Yes | Yes | Yes |
| 4. Were the subjects comparable? | No | No | No | No | No | No | No | No | No |
| 5. Was the intervention clearly described? | Yes | Yes | Yes | Yes | Yes | Yes | Yes | Yes | Yes |
| 6. Were the outcome measures clearly defined, valid, reliable, and implemented consistently across all study participants? | Yes | Yes | Yes | Yes | Yes | Yes | Yes | Yes | Yes |
| 7. Was the length of follow-up adequate? | Yes | Yes | Yes | Yes | Yes | NR | Yes | Yes | Yes |
| 8. Were the statistical methods well-described? | Yes | Yes | Yes | Yes | Yes | No | No | No | Yes |
| 9. Were the results well-described? | Yes | Yes | Yes | Yes | Yes | Yes | Yes | Yes | Yes |

| **Criteria** | **Studies** | |
| --- | --- | --- |
|  | Wen 1994  [46] | Nagakawa 1993 [37] |
| 1. Was the study question or objective clearly stated? | Yes | Yes |
| 2. Was the study population clearly and fully described, including a case definition? | Yes | Yes |
| 3. Were the cases consecutive? | Yes | Yes |
| 4. Were the subjects comparable? | No | No |
| 5. Was the intervention clearly described? | Yes | Yes |
| 6. Were the outcome measures clearly defined, valid, reliable, and implemented consistently across all study participants? | Yes | Yes |
| 7. Was the length of follow-up adequate? | Yes | Yes |
| 8. Were the statistical methods well-described? | No | Yes |
| 9. Were the results well-described? | Yes | Yes |

**Table 2.** Supplementary procedural data

| **Study** | **Earliest PP-QRS interval during FVT (ms)** | **Fascicular block after ablation % (N)** |
| --- | --- | --- |
| Ma 2017  [38] | 34.1±4.2  24.5±3.1  19.4±2.8 | 15% (6) |
| Luo 2017 [24] | N/A | 93.66% (123) |
| Guo 2017 [13] | 35.9±4.5 | 20% (2) |
| Zan 2016 [25] | N/A | 0% (0) |
| Liu Q 2016 [8] | N/A | 0% (0) |
| Chen 2016 [19] | 34±9.4 (LAFT)  23.5±6.8 (LPFT) | 0% (0) |
| Liu Y 2015 [7] | 30.1±9.3 | 20% (23) |
| Letsas 2015 [39] | 21.3±3.7 | 0% (0) |
| Gopi 2015 [14] | N/A | 0% (0) |
| Fishberger 2015 [22] | N/A | 0% (0) |
| Talib 2015 [17] | N/A | 0% (0) |
| Liu XY 2014 [26] | N/A | 0% (0) |
| Suzuky 2014 [48] | N/A | 17% (1) |
| Collins 2013 [15] | N/A | 0 (0%) |
| Kataria 2013 [20] | 12±1.7 | 0% (0) |
| Park 2012 [40] | 22.2±8.2 | 0% (0) |
| Wissner 2012 [41] | N/A | 0% (0) |
| Chu 2012 [27] | N/A | 0% (0) |
| He 2011 [28] | 34.9±13.1 | 0% (0) |
| Shin 2010 [21] | 21.2±8.4 | 0% (0) |
| Ma 2006 [42] | 26.3±4.6 | 100% (39) |
| Magalhaes 2006 [16] | 31±20.4 | 0% (0) |
| Lin 2005 [43] | N/A | 34% (2) |
| Arya 2004 [29] | 18±4 | 0% (0) |
| Topilski 2004 [18] | N/A | 0% (0) |
| Gupta 2002 [44] | N/A | 0% (0) |
| Ouyang  2002 [30] | 14.8±6.5**‡** | 0% (0) |
| Aiba 2001 [31] | 13±7 | 0% (0) |
| Miyauchi 2000 [32] | 20±8.7 | 0% (0) |
| Nogami 2000 [33] | 14±10 | 0% (0) |
| Tsuchiya 1999 [34] | 15.2±9.6 | 0% (0) |
| Nogami 1998 [35] | 61.7±.5.1* | 0% (0) |
| Bennett 1997 [47] | N/A | 0% (0) |
| Wen 1997 [45] | 13.7±.4.6 | 0% (0) |
| Katristis 1996 [23] | N/A | 0% (0) |
| Zardini 1995 [36] | 13.7±.4.1 | 0% (0) |
| Wen 1994 [46] | N/A | 0% (0) |
| Nagakawa 1993 [37] | 27±9 | 0% (0) |
| **Total/Mean** | **-** | **20.6% (196)** |

**Abbreviations**: N/A: non-available; FVT: fascicular ventricular tachycardia; PP: Purkinje potential. **Note**:

* data available for 3 patients only; **‡** available for 6 patients.
